# Impact of government’s dual-carbon attention on urban carbon emissions reduction: evidence from the Yangtze River Delta

**DOI:** 10.1038/s41598-025-03541-3

**Published:** 2025-10-23

**Authors:** Kai Chang, Jing Li, Shuqi Wei, Boyang Li

**Affiliations:** 1 School of Finance and Business, Shanghai Normal University, Shanghai, 200234 China; 2 School of Data Science and Engineering, East China Normal University, Shanghai, 200241 China

**Keywords:** GCA, Carbon emissions, Industrial structure upgrading, Emission-reduction policies, STIRPAT model, Behavioural ecology, Climate-change ecology, Environmental economics

## Abstract

There is an ever-increasing concern among local governments in China regarding the relationship between government attention and carbon emissions. This study proposes a new dual-carbon attention dictionary and measures local governments’ dual-carbon attention (GCA) level using textual analysis and the maximum reverse matching method. Our research highlights that an extended STIRPAT model exhibits the significance of local governments’ efforts in achieving CO_2_ emissions reduction. Empirical evidences demonstrate that increased GCA leads to a greater reduction in urban CO_2_ emissions. Furthermore, GCA greatly decreases urban CO_2_ emissions in the Yangtze River Delta (YRD) via accelerating the upgrading of industrial structures and improving emission-reduction policies implementation and reducing carbon emissions intensity. Additionally, the GCA has stronger robustness in decreasing urban CO_2_ emissions in the YRD under peers GCA, environmental governance policies and environmental protection investment.

## Introduction

On September 22, 2020, the Chinese government presented its dual-carbon targets at the 75th United Nations General Assembly. These targets involve making significant efforts to achieve a peak in CO_2_ emissions by 2030 and attaining carbon neutrality by 2060. The Fifth Plenary Session of the 19th Central Committee of the Communist Party of China identified key areas for achieving the following targets. They include promoting green technological innovation, encouraging the adoption of green production methods and sustainable lifestyles, implementing comprehensive green transformations in economic and social development, and achieving stable and moderate reductions in carbon emissions after reaching the peak by 2030. In April 2021, the 14th Five Year Plan prioritizes improvements in environmental quality, comprehensive enhancements in resource utilization efficiency, and the promotion of harmonious coexistence between humans and nature. The report of the 20th National Congress of the Communist Party of China, released on October 25, 2022, further emphasized the intensified efforts to prevent and control environmental pollution, actively and steadily advance the achievement of carbon emissions peak and carbon neutrality, and foster the development of a green economy.

Local governments bear significant responsibilities for various governance tasks, including economic development, social management improvement, ecological environment governance, and the achievement of dual-carbon targets. The concept of local governments’ dual-carbon attention (GCA) prompts local government decision-makers to prioritize crucial issues related to environmental governance, dual-carbon governance resources, including seven dimensions, such as carbon emissions peaking, carbon emissions neutrality, energy conservation, emission reduction, ecological environment governance, green finance sustainable development as well as dual-carbon technology implications. In this study, GCA not only includes dual-carbon governance achievement, but also includes crucial six issues, such as environmental governance and energy-saving and emission-reduction issues in the 11 ~ 13th Five Plan. These GCA issues represent a concentrated reflection of the transfer and allocation of crucial resource as well as technology progress and policy supports.

In order to ensure the effective completion of dual-carbon governance tasks, local governments are urgently strengthening their continuous GCA in government work reports. The effectiveness of dual-carbon governance is closely linked to GCA issues. Due to their own interests and external institutional constraints, different local governments may prioritize different policy attributes and exhibit varying motivations for interest-driven behavior when understanding policy signals from the central government. GCA issues serves as an important factor influencing government decision-making behavior and the overall process of environmental governance. It significantly impacts the effectiveness of dual-carbon and environmental governance.

In the YRD region, Shanghai, Zhejiang Province, and Jiangsu Province are jointly working on the planning and implementation of the YRD Ecological and Green Integrated Development Demonstration Area. Compared to other urban agglomerations in China, the YRD urban agglomeration is extremely important socio-economic and political contexts. The YRD urban agglomeration is the largest urban group in China with the greatest economic development engine and faster factor flow, aiming to fully build a world-class urban agglomeration with global influence by 2030. Based on the “2023 Yangtze River Delta Region Climate Change Action Report”, the total GDP of the YRD urban agglomeration is 29.03 trillion RMB in 2022,, accounting for about a quarter of China’s GDP. In 2020, the energy consumption intensity per unit of GDP decreased by 18% compared to 2015, and the carbon emissions intensity per unit of GDP decreased by about 22%. Therefore, exploring the relationship and influence mechanism between GCA and urban emissions reduction is an extremely critical issue, which has important theoretical and practical value for exploring other urban agglomerations.

The government work report (GAR) is a key policy document that signals the issues that government at different levels deems important. It serves as crucial information that local governments convey to the public and stakeholders^1^. The annual consolidated report for local governments summarizes and evaluates the anticipated as well as unanticipated social and economic outcomes of key information in public administration^2,3^. The publication of information on sustainability issues by local governments conveys the relationship between sustainability information disclosure and various socioeconomic, financial, and economic factors^4,5^. Generally, government work reports pay much attention to important categories: macro-economic issue and citizen livelihood issue and other issues.

The local government’s environmental attention is a crucial and limited resource that signifies the level of focus given by government decision-makers to specific affairs or issues. Changes in government environmental attention are indicative of shifts in decision-making choices, resource allocation, and the effectiveness of environmental governance. Government environmental attention exerts a lagged effect on reducing regional air and environmental pollution, and then improving environmental governance via environmental laws and green investment^6–9^. Government environmental attention promotes urban energy efficiency and carbon emission reduction^10,11^. Increased government environmental attention promotes firm green innovation via environmental regulation and green investment^12,13^. Government environmental attention notably reduces firms’ carbon emissions via environmental regulations, fiscal policies, and energy structure transformation^14,15^. Government environmental attention facilitates firms’ social responsibility and ESG performance via green investment and green innovation^16,17^.

The ecological and environmental governance of local governments plays a crucial role in mitigating energy intensity and environmental governance through the implementation of green development initiatives and industrial upgrading strategies^18,19^. Media attention may influence policy response to curbing greenhouse gas emissions^20^. Private firms operating in high-carbon industries may experience a higher sensitivity to and lower tolerance for environmental regulations when they are subjected to high positive media attention^21^. Media attention may weaken the influence of the impression management of firm’s carbon information disclosure with external financing demand^22^. Climate change attention produces important guidelines for investment and policy-making in environmental protection^23^.

Government intervention and policy implementation play significant roles in achieving carbon emission reduction and promoting sustainable transformation. By implementing policy regulations, the government intervention and regulation influences the low-carbon policy-making and energy-saving decisions, thereby produces an influence on carbon emission reduction and carbon decoupling efforts^24,25^. Environmental regulations have the potential to impact carbon emissions through various mechanisms, including promoting technical efficiency improvements, influencing energy consumption patterns, and shaping industrial structures^26–29^. Environmental regulations aim to limit local manufacturing carbon emissions. However, environmental competition enhance their environmental regulatory performance, optimize industrial structures, and thus improve energy efficiency and carbon emission reductions^16,30,31^. The combination of market segmentation and government environmental intervention has a significant inhibitory effect on carbon emission intensity by promoting technical innovation^32,33^. Strengthening environmental regulations and reducing dependence on natural resources are to effectively control environmental pollutions and reduce carbon emissions^34,35^.

The implementation of carbon emissions trading policies has been recognized as a significant institutional innovation that promotes sustainable and low-carbon development^36^. Numerous studies have found that carbon trading policies effectively increase environmental investments and encourage carbon emissions reduction through energy consumption^37–39^. Carbon emissions trading pilots have been shown to generate environmental dividends, facilitate technological progress, and reduce carbon emissions for firms^40–43^. European environmental policies have contributed to decreased carbon dioxide emissions for firms regulated under the European Union Trading Scheme, impacting their future valuations^44,45^. Rigorous environmental regulation policies also enhance total factor energy productivity and improve consumption-based carbon emissions efficiency across industries through industrial restructuring and technical innovation^46–48^. Additionally, environmental regulations have been found to enhance carbon emissions reduction through technical innovation^49–51^. China’s environmental policies have significantly improved carbon emission efficiency by reducing energy consumption intensity and upgrading industrial structures^52,53^. While the stringency of environmental policy instruments is associated with increased carbon emissions in middle-income economies^54^, revenue from taxation, stringent environmental policies, and the promotion of renewable energy sources have been found to mitigate CO_2_ emissions^55^. Furthermore, there is evidence to show that the synergy of environmental regulation policies exhibits a significant N-shaped effect on carbon emissions^56^.

The consistent focus of local governments on the ecological environment is a crucial factor that impacts regional energy intensity and investment in environmental pollution control. Local governments play a significant role in curbing environmental pollution by directing their attention towards green development and industrial upgrading. However, due to limited resources and bounded rationality in government decision-making, it is imperative for government decision-makers to address the following challenges: how to redirect local governments’ attention towards energy-saving and emission reduction, enhance policy implementation, improve green productivity, promote industrial structure transformation, and establish an effective path towards dual-carbon transformation and governance efficiency.

Firstly, the GCA concept is abstract and necessitates the development of scientific methodologies to convert intangible political resources into tangible data. GCA is a continuous and dynamic evolutionary trend, providing the basis for scientifically identifying local governments’ allocation decisions and the rules governing attention resources in relation to dual-carbon governances.

Secondly, it is crucial to examine whether the GCA can promote the upgrading of industrial structures and enhance the potential for emission reduction. Furthermore, the effectiveness of local governments in creating efficient transmission channels for energy-saving and emission reduction needs to be explored.

Our study contributes to the existing literature on the ecological environment by examining the consequences of government attention^6–11^ and identifying multidimensional policy influences. Our research makes three key contributions:

First, we propose a novel theoretical framework for understanding the influence of GCA, using a textual analysis method to measure the frequency of dual-carbon keywords from seven dimensions, including carbon emission peak, carbon emission neutralization, green finance, ecological environmental governance, energy-saving and emission reduction, sustainable development, and lower-carbon technology implications. This approach provides a comprehensive measurement of GCA. We expand the newly developed STIRPAT model by incorporating population, economic factors, technology, government attention, and urbanization. Our findings highlight the importance of GCA in reducing urban carbon emissions in the YRD cities.

Second, our research emphasizes the significance of interactions and emergent effects in reducing urban CO_2_ emissions outcomes. Our study demonstrates that GCA has significant decreasing effects on urban carbon emissions in the YRD region by promoting industrial structure upgrading, reducing carbon emission intensity and improving emission-reduction policies implementation. These findings contribute to the understanding of the complex relationship between local governments and environment governance, and provide newly insights for shaping effective government dual-carbon policies and enhancing environmental governance.

Third, the peers GCA, environmental governance policies and environmental protection investment yet results in the rapid reduction of urban CO2 emission in the YRD region. Additionally, robustness results demonstrate that the GCA enlarges the reduction effects on urban CO_2_ emissions in the YRD region under the above conditions.

Overall, our research enriches understanding of the role of government attention and provides a framework for comprehending the intricate linkages between local government and ecological environment governance. This study informs policymakers and raises awareness regarding the effectiveness of environmental governance measures.

The subsequent sections are structured as follows: Section “Theoretical analysis and research hypothesis” provides an overview of the main theoretical analysis and research hypotheses. The variables selection and the expanded STIRPAT model are presented in Section “Variable selection and research method”. Section “Data source and statistical description” outlines the data source and examines the evolution trend of governments’ double-carbon attention. Section “Empirical results and discussions” explores the direct and indirect influence of double-carbon attention on urban carbon emissions. Finally, Section “Conclusions and policy implications” summarizes the main conclusions drawn from the study.

## Theoretical analysis and research hypothesis

Local government attention is a scarce and limited social resource. In recent years, local governments gradually strengthen their dual-carbon issues such as greening, low-carbon, sustainable development, environmental governance, ecological protection, energy saving and emission reduction, carbon peak, and carbon neutrality in the annual GWR reports. Local government decision-makers are shifting more social resources towards ecological environment and dual-carbon governance, and sustainable development tasks. Local government’s competition, incentive systems, and technological innovation are catching up to accelerate urban dual-carbon governance. Local governments strengthen of these important issues of dual-carbon governance and shift towards low-carbon development, ecological environment and dual carbon governance, and thus reduce urban CO_2_ emissions.

The central government establishes an effective constraint and assessment mechanism in energy saving and emission reduction, ecological environment governance, energy dual-control system, and pollutant control targets. These assessments are linked to the promotion of government officials and encourage local government officials to strengthen their attention to dual-carbon governance. The “Promotion Championship Theory” holds that local governments actively achieve specific economic growth and dual- carbon governance targets based on the “benchmark competition” of government performance assessment^14,15^. These tasks strengthen the intervention preference for economic development and ecological governance, demonstrating the loyalty and signaling ability of government officials towards the dual-carbon tasks^57^. Under the official promotion tournament, local governments strive for a great amount of economic and social resources, implement effective policies and measures for energy saving and emission reductions, ecological environment and dual-carbon governance in order to achieve the expected dual- carbon governance tasks. Local governments adapt incentive systems such as economic incentives and financial supports to motivate firms to improve energy saving and emission reduction efficiency, and thus accelerate the potential to reduce urban carbon emissions.

Local governments strengthen their low-carbon technology innovation and catch-up strategies, such as energy-saving and emission reduction technologies, renewable energy technologies, and carbon neutrality technologies. They are actively learning, tracking, imitating, absorbing, and applying advanced low-carbon technology and independent innovation efforts and thus accelerate the reduction of urban carbon emissions. The Porter hypothesis suggests that GCA is beneficial for local governments to optimize green innovation systems, accelerate the transfer of industries to high-end industries^7,8^. Many firms enhance their ability to absorb green technologies, and improve their ability to green production. The advancement of energy-saving and emission reduction technologies promotes firms to achieve green innovation efficiency, accelerates the reduction of urban carbon emissions, and creates greater innovation compensation effects.

### Hypothesis 1 GCA decreases urban CO_2_ emissions

The dual-carbon governance necessitates novel demands on local governments. These demands encompass ensuring an overall industrial layout, facilitating the low-carbon transformation of energy systems, and promoting the potential of urban CO_2_ emissions. Local governments expedite the upgrading of industrial structures and the transformation of energy consumption patterns towards cleaner energy^58^. The increase in the secondary sector is a significant driver of urban energy consumption and carbon emissions. In contrast, the service sector exhibits relatively lower energy consumption per unit of output. Therefore, local governments prioritize the development of the service industry and foster industrial structure upgrading, and result in a considerable reduction in energy consumption and carbon emissions.

Government decision-makers are increasingly attentive to the redistribution and allocation of resources among industries. Their efforts focus on facilitating the transition of high energy-consuming and pollution-intensive industries towards low-carbon service sectors^59^. GCA can improve the allocation of resources among various industries, resulting in a more sustainable investment. Local governments optimize a larger share of energy and resources towards sustainable and low-carbon industries, as well as high-end manufacturing and service sectors.

Local governments actively prioritize sustainable technological transformation and the efficient allocation of resources across industries, as well as the rapid upgrading of the industrial structure^60,61^. The government decision-makers allocate a greater number of social resources towards the upgrading of industrial structure, and concurrently promote energy-saving and emission reduction in both manufacturing and service industries and thus enhance urban energy efficiency. Local governments establish and refine industrial policies, and continually optimize and correct the industrial structure. The upgrading of the industrial structure can promote urban resource allocation efficiency and ecological management efficiency.

Local governments actively develop the renewable energy industry, reduce the consumption structure of fossil fuels, and thus promote low-carbon transformation of urban energy system. These initiatives contribute to sustainable industrial development and the overall efficiency improvement of dual-carbon governance. Consequently, they elevate the ability of ecological environment regulation and impose stricter entry thresholds for industries. Additionally, local governments vigorously introduce green industries and strategic emerging industries, mobilize a greater number of social resources to bolster green industries such as energy-saving and environmental protection, clean production, clean energy, and ecological environment. Accelerating the rationalization of the industrial structure and promoting its transformation towards high-end industries are essential steps to reduce urban CO_2_ emissions.

Hypothesis 2: The GCA has a significant impact on decreasing urban carbon emissions via facilitating industrial structure upgrading.

Local governments undertake efforts to transform the energy consumption and economic development modes, strategically reduce the growth of fossil energy consumption and high pollution industries, and enhance policies and measures that promote industrial restructuring. Local governments emphasize major industrial structure upgrading like manufacturing, construction, transportation, public utilities, and ecological construction, strengthen monitoring activities of ecological environment and urban CO_2_ emissions, and increase investment and supervision in environmental governance. They actively seek to coordinate pollution and CO_2_ emissions governance, reduce urban water, and solid waste pollutants as well as greenhouse gas emissions. Local governments are progressively enhancing the energy dual-control system and policy framework to effectively reduce the total amount and intensity of energy consumption, and thus decrease urban CO_2_ emissions^62^.

Furthermore, local governments develop and reinforce renewable and low-carbon energy sources, promote industrial restructuring and energy consumption structure optimization, optimize energy resource allocation efficiency and CO_2_ emissions efficiency. GCA inputs a great amount of resources and uses renewable energy towards accelerating dual-carbon governance tasks. GCA facilitates the efficient utilization of energy resources across industries and effectively reduces energy consumption intensity and CO_2_ emissions intensity, and finally decreases urban CO_2_ emissions.

Local government decision-makers argue that carbon-intensive and pollution-intensive industries are the primary sources of urban CO_2_ emissions intensity. Local governments strengthen the alignment of policies with industrial structure, optimize the allocation of factor resources, and promote the development of regional industrial structures that are low-carbon, high-end, and rationalized. These efforts make greater reductions in urban energy consumption intensity and CO_2_ emissions intensity, and thus decrease urban CO_2_ emissions.

Moreover, local government decision-makers recognize that the high proportion of fossil energy consumption is a significant obstacle to achieving urban CO_2_ emission reduction. Therefore, local governments make increased efforts to break technological barriers and dependencies on fossil energy consumption, and thus promote urban green innovation capacities^63^. Local governments are actively developing renewable energy sources, promoting the low-carbon transformation of the energy system, and gradually establishing a dual-substitution of energy structure. Electrification replaces coal and renewable energy replaces fossil fuels, and thus facilitates the substitution of energy structure and reduces CO_2_ emissions intensity.

Hypothesis 3: Increased GCA results in greater reduction of urban CO_2_ emissions via decreasing carbon emissions intensity.

Both the central and local governments place significant importance on energy conservation and emissions reduction. In 2007, the State Council of China issued a notice on the comprehensive work plan for energy saving and emission reduction, which set strict constraint targets for unit GDP energy consumption, industrial water consumption, major pollutant emissions, sewage treatment, and solid waste treatment etc. In the subsequent Five-year Development Plan and annual Government Work Reports, energy-saving and emissions reduction targets, as well as the control of major pollutants, are regarded as crucial indicators to reinforce the government’s responsibility and the responsibility of market entities. During the period from the 11th Five Year Plan to the 13th Five Year Plan, local governments have become mandatory institutional arrangements to promote the development of industry and service industries.

Local governments increase their attention to energy saving and emission reduction targets, gradually strengthen important issues such as ecological environment governance, energy saving and emission reduction technologies, emission reduction potential, carbon peak, carbon neutrality, etc. These issues enhance the execution power of local governments in providing energy saving and emission reduction policies. Local governments input resources to promote the progress of energy-saving and emission reduction technologies in the industrial and service industries. These technologies incentivize firms to accelerate industrial clean production, improve energy efficiency, and enhance the implementation of urban energy-saving and emission reduction policies. The GCA establishes standardized policy systems and implements economic incentives to promote the implementation of energy-saving and emissions reduction.

The GCA enables local governments to establish standardized and comprehensive institutional mobilization and incentive capacities for both local government departments and market entities. The GCA gradually forms an intergovernmental normative policy process innovation, establishes a collaborative decision-making and governance model for energy-saving and emission reduction. The greater implementation of energy-saving and emission reduction policies forces local governments to focus their attention on important issues such as energy-saving and emission reduction technologies, energy efficiency, and ecological environment governance, in order to improve energy-saving and emission reduction efficiency. Strengthening the GCA can help improve the implementation of energy saving and emission reduction policies, and thus reduce urban CO_2_ emissions. This positive impact is mainly reflected in two aspects: on the one hand, local governments implement energy-saving and emission reduction policies, coordinate government’s and firm’s emission-reduction actions through various energy-saving and emission reduction policies measures. They promote resource concentration and improve the implementation of energy-saving and emission reduction policies, and improve urban energy-saving and emission reduction efficiency. On the other hand, local governments continuously strengthen GCA, increase the pressure of performance evaluation for energy saving and emissions reduction, and accelerate their policies implementation, which accelerate the decline of urban CO_2_ emissions.

Hypothesis 4 GCA decreases urban CO_2_ emissions via improving the implementation of emission-reduction policies.

**Fig. 1:**
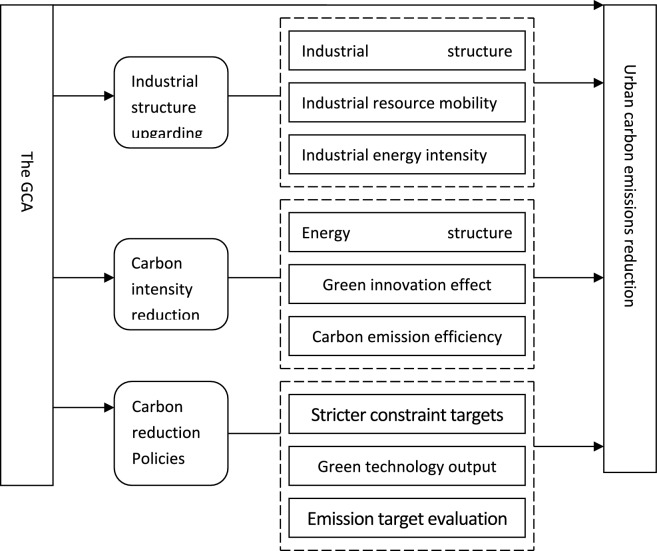
The influence mechanism between GCA and urban CO_2_ emissions.

And Fig. 1shows the influence mechanism between GCA and urban CO_2_ emissions as follows.

## Variable selection and research method

### Variables selection

The dependent variable in this study is urban CO_2_ emissions, which is measured as the natural logarithm of a city’s CO_2_ emissions. The data for CO_2_ emissions in cities located in the Yangtze River Delta region were obtained from the China Carbon Accounting Database, which was developed by the research team led by Guan Dafu from Tsinghua University in 2016. This database includes CO_2_ emissions data from 47 socio-economic sectors, as well as 17 processes related to fossil fuel combustion and cement production. The carbon dioxide emission accounting method used follows the guidelines recommended by the Intergovernmental Panel on Climate Change (IPCC), which encompasses all direct carbon emissions resulting from human socio-economic activities within the urban administrative boundary.

**Fig. 2:**
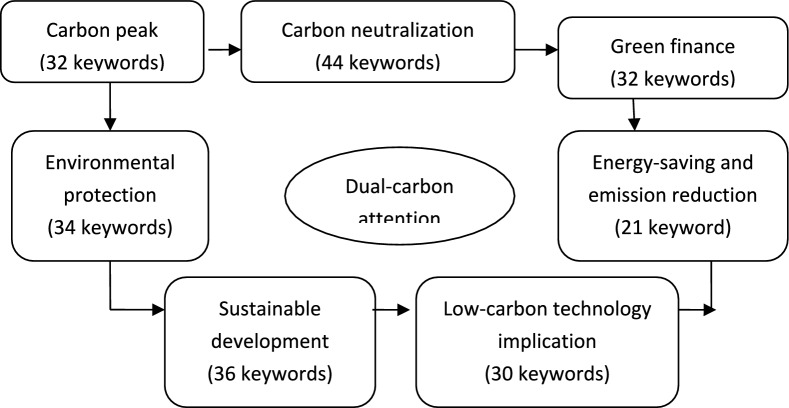
The specific dual-carbon attention flowchart of local governments.

The independent variable in this study is the GCA level. This variable is measured as the natural logarithm of the total frequency of dual-carbon related words extracted from the annual GWR texts in the YRD cities. In the robustness test, this study adapts the GCA substituted by GCA intensity. The process of measuring the dual-carbon attention allocation involves five main steps as outlined below:

Firstly, a dictionary of dual-carbon attention is constructed. This study collects GWR texts from 41 prefecture-level cities in the YRD region during the period from 2005 to 2019. Additionally, policies texts related to dual-carbon transformation are obtained from the magic weapon database of Peking University. A thorough analysis of these policies texts is conducted by our research team to build a vocabulary corpus specifically focused on dual-carbon attentions. After careful deliberation and consultation with industrial experts, the study team identifies seven dimensions for the dual-carbon transformation dictionary, namely carbon peak, carbon neutrality, green finance, energy-saving and emission reduction, environmental governance (environmental protection), green (sustainable) development, and dual-carbon technology application. Ultimately, a comprehensive dictionary consisting of 229 keywords related to dual-carbon attention is derived. The detailed dual-carbon dictionary can be found in (Appendix Table 1), and Fig. 2 presents a visual representation of the specific dual-carbon attention flowchart of local governments.

**Table 1:** Selection and definition of related variables.

Variable type	Variable name	Variable symbol	The definition of related variables
Explained variable	CO_2_ emissions	$I_{it}$	Urban CO_2_ emissions
Explainary variable	GCA	$GCA_{it}$	The sum of all the word frequencies of double-carbon keywords from the annual GWR
	GCA intensity	$GCAI_{it}$	The total dual-carbon word frequency*100/total words of GWR texts
Mediating variables	Industrial structure upgrading	$UIS_{it}$	value added of the tertiary industry/value added of the secondary industry
	Carbon emission intensity	$CI_{it}$	carbon emissions amount/GDP
	Emissions reduction policies implementation	$SERI_{it}$	The difference between the actual and targeted SO_2_ emission intensity
Control variables	Economic development	$A_{it}$	Urban per capita GDP
	Population size	$P_{it}$	urban permanent population
	Energy consumption intensity	$EI_{it}$	electricity consumption amount/urban GDP
	Urbanization level	$UD_{it}$	The number of urbanized population/the number of total urban population

Secondly, the GWR texts are transformed using GROBID, which is a machine learning library known for its ability to automatically extract, parse, and organize information from raw PDF documents, converting them into structured XML/TEI encoded files. In this study, a Python text-mining program was developed to utilize the GROBID library for batch converting the PDF text into the annual GWR texts (in txt format) specifically pertaining to the cities in the YRD region.

Thirdly, the text pre-processing step is carried out. It is important to note that the GWR texts processed by GROBID might have some truncated words due to internal line breaks. Consequently, these truncated words may contain crucial keywords related to the double-carbon attention. To enhance the accuracy of word frequency statistics related to double-carbon attention keywords, regular expressions are employed to automatically remove line breaks, numbers, letters, underscores, hyphens, and stop words from the GWR txt texts. This process aims to obtain a cleaned corpus of the GWR text data.

In the fourth step, this study utilizes the “jieba” software package in Python for automatic segmentation of the GWR txt texts from cities in the YRD region. Subsequently, a text mining program focused on dual-carbon attention is developed, utilizing textual information analysis and the maximum reverse matching method. This program facilitates the automatic extraction of the total frequency of double-carbon attention keywords from the GWR texts.

Lastly, the research team gathers the total word frequency of dual-carbon attention from the corpus of the GWR texts related to the cities in the YRD region. The natural logarithm of their total word frequency is then employed as a measure to quantify the GCA level. The GCA intensity is measured to the total dual-carbon word frequency*100, divided by the total words of GWR texts.

To further investigate the underlying factors influencing GCA, this study incorporates industrial structure upgrading, carbon emission intensity and emission-reduction policies implementation as mediating variables. Industrial structure upgrading is assessed through the ratio of added value in the tertiary industry to added value in the secondary industry. Carbon emission intensity is estimated by dividing urban CO_2_ emissions by GDP, and energy consumption intensity is obtained by dividing urban electricity consumption by GDP. SO_2_ emission-reduction policies implementation is defined as the difference between the actual SO_2_ emission intensity ratio and the targeted SO_2_ emission intensity ratio. Its specific formula is written as:

1 $$SERI_{it} = ( - 1) \times \frac{{CI_{it} - CI_{i(t - 1)} }}{{CI_{i(t - 1)} }} - ( - 1) \times TCR_{it}$$

Here $SERI_{it}$ denotes SO_2_ emission-reduction policies implementation, $TCR_{it}$ denotes the targeted SO_2_ emissions intensity ratio. SO_2_ emissions intensity is defined as the ratio of urban SO_2_ emissions divided by urban GDP. To account for the various influences of GCA on urban CO_2_ emissions, this study considers the related control variables, such as population size, economic development, technology progress, urbanization level. Consequently, an expanded STRIPAT model is developed. The precise definitions of these related variables are outlined in (Table 1).

## Research method

In order to enhance local governments’ efforts in reducing urban CO_2_ emissions, they focus on several driving factors. These include optimizing and adjusting the urban industrial structure, upgrading the industrial sector, reducing energy consumption intensity and carbon emission intensity, and the quality of economic development etc. These measures are implemented by strengthening the implementation of energy-saving policies. Based on these considerations, this article examines the effects of GCA on urban CO_2_ emissions by selecting industrial structure upgrading, carbon emission intensity, emission reduction policies implementation as mediating factors. To analyze the causal relationship between carbon emissions and driving factors such as population scale, economic development, and technology level, the authors employ the IPAT model proposed by Ehrlich and Holdren^64^. The specific expression is as follows:

2 $$I = a \times P^{b} \times A^{c} \times T^{d} \times e$$

In Eq. (2), I represents the urban CO_2_ emissions of environmental pressure factors, P, A and T represents the factors that affect the population scale, economic development and technical level of CO_2_ emissions, respectively. $a$ represents the model constant coefficient, $b,c,d$ are the coefficients of the above mentioned impact factor, and $e$ is the error term of the model. In order to facilitate the measurement of the direction and degree of influence of various influencing factors, we adapt the following expression in empirical analysis:

3 $$\ln I_{it} = a + b\ln P_{it} + c\ln A_{it} + d\ln T_{it} + \xi_{it}$$

However, to overcome the limitations of the IPAT model, Dietz and Rose (1997) propose an expanded STIRPAT model for practical application^65^. To better capture the specific characteristics of the YRD cities, this study extends the analysis to explore the potential impact of GCA on urban carbon emissions. Several key variables are considered, including population scale, per capita GDP, energy consumption intensity, urbanization level, and the GCA level. These variables are incorporated into a new STIRPAT model as follows:

4 $$\ln I_{it} = a + b\ln P_{it} + c\ln A_{it} + d\ln T_{it} + e\ln GCA_{it} + f\ln UD_{it} + \xi_{it}$$

In Eq. (4), $\ln GCA_{it} ,\ln UD_{it}$ refers to the natural logarithm of the GCA and urbanization level in the YRD cities. To examine the impact of GCA on urban CO_2_ emissions, this study considers several mediating variables: industrial structure upgrading, carbon emission intensity, and the implementation of emission-reduction policies. These variables are selected to assess the influence channels of GCA on CO_2_ emissions reduction. And their economic models are expressed as follows:

5 $$\ln X_{it} = {\text{a}} + b\ln P_{it} + c\ln A_{it} + d\ln T_{it} + e\ln GCA_{it} + f\ln UD_{it} + \xi_{it}$$

6 $$\ln I_{it} = a + b\ln P_{it} + c\ln A_{it} + d\ln T_{it} + e\ln GCA_{it} + g\ln X_{it} + f\ln UD_{it} + \xi_{it}$$

Here $X_{it}$ refers to industrial structure upgrading, carbon emission intensity and the SO_2_ emission-reduction policies implementation. Equation 5 demonstrates the GCA produces the significant impact on the above mediating variables. Equation 6 confirms that both the GCA and mediating variables produce the significant effect on urban CO_2_ emissions reduction.

## Data source and statistical description

### Data source and measurement

In December 2019, the Outline of the Regional Integration Development Plan in the Yangtze River Delta expanded the coverage of the YRD cities to include 41 low-level cities located in Shanghai, Jiangsu (13 cities), Zhejiang (11 cities), and Anhui (16 cities) provinces. The YRD region is characterized by rapid economic development, substantial potential for energy conservation and emission reduction, as well as a notable decoupling of economic growth and energy consumption. Given the dual-carbon target, the YRD cities hold significant importance as they are well-positioned to achieve the carbon peak target in a timely manner. This achievement not only supports the Chinese government in realizing the dual-carbon target but also sets a noteworthy example for other cities aiming to achieve the same target.

This study gathers panel data from the annual government work report texts of 41 prefecture-level cities in the Yangtze River Delta region. The data is obtained from official government websites and the Peking University Fabao Database. To ensure the consistency and completeness of the government work report (GWR) text data, the sample of cities covers the period from 2005 to 2019. In total, 615 GWR reports from prefecture-level cities were collected for the study.

The GCA level is calculated using textual big-data mining technology, which involves crawling and analyzing the frequency of keywords related to dual-carbon from the annual GWR texts. CO_2_ emissions data for the prefecture-level cities are sourced from the China Carbon Accounting Database. Urban CO_2_ emissions covered the period from 2005 to 2019. The assessment of emission reduction policies is based on the difference between the target ratio and the actual ratio of emission reduction achieved by the State Council during the 11th, 12 and 13th Five-Year Plan periods. Other pertinent data primarily come from the Economy Prediction System (EPS) data platform and the China Urban Yearbook.

### Descriptive statistics of related variables

**Fig. 3:**
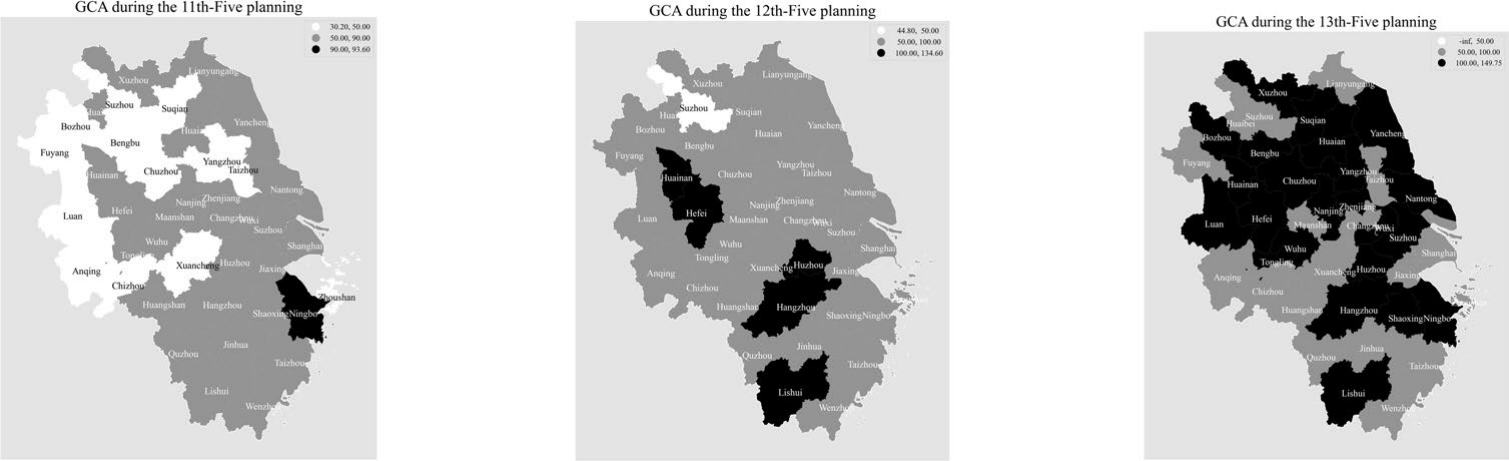
The trends of GCA levels in the YRD cities during the11, 12 and 13th five-year plan.

Table 2 and Fig. 3 illustrate the average trends of GCA in the Yangtze River Delta cities during the 11 h, 12th and 13th Five-Year Plans. This study categorizes the total word frequency of GCA into three levels: 50–90, 90–123, and 124–180. The map data is sourced from Alibaba Cloud Data Visualization Platform—Gaode Open Platform https://datav.aliyun.com/portal/school/atlas/area_selector. Figure 2 is to use Matplotlib instrument of Python software.

**Table 2:** Statistical trends of GCA in the YRD cities during the 11, 12 and 13th five-year plans.

City	11th	12th	13th	City	11th	12th	13th
Shanghai	84.2000	90.4000	96.2500	Shaoxing	68.2000	67.0000	107.5000
Nanjing	61.6000	95.2000	128.2500	Zhoushan	48.2000	60.6000	62.5000
Nantong	70.6000	80.6000	119.2500	Quzhou	60.0000	70.0000	92.5000
Suqian	34.0000	65.0000	122.5000	Jinhua	63.2000	79.6000	86.2500
Changzhou	61.4000	70.0000	100.0000	Bozhou	30.8000	67.0000	103.7500
Xuzhou	58.4000	76.2000	115.7500	Lu’an	45.6000	87.3333	100.7500
Yangzhou	49.0000	80.4000	112.0000	Hefei	60.6000	114.0000	112.7500
Wuxi	67.4000	87.0000	106.2500	Anqing	40.6667	74.2000	90.5000
Taizhou^①^	46.8000	58.6000	89.2500	Xuancheng	30.2000	61.4000	97.7500
Huaian	82.0000	56.8000	108.7500	Suzhou^④^	31.4000	44.8000	89.0000
Yancheng	63.0000	88.4000	118.2500	Chizhou	46.2000	67.7500	96.3333
Suzhou^③^	80.8000	94.6000	122.0000	Huaibei	54.4000	63.6000	77.3333
Lianyungang	61.6000	75.0000	90.7500	Huainan	78.5000	100.8000	107.2500
Zhenjiang	55.4000	56.8000	101.5000	Chuzhou	49.6667	83.0000	101.0000
LiShui	81.0000	113.4000	149.7500	Wuhu	53.4000	74.6000	106.0000
Taizhou^②^	65.0000	95.2000	71.5000	Bengbu	40.0000	66.4000	107.2500
Jiaxing	73.0000	92.8000	83.0000	Tongling	62.0000	75.8000	114.5000
Ningbo	93.6000	92.6000	101.2500	Fuyang	39.6000	54.6000	96.2500
Hangzhou	83.2000	134.6000	107.7500	Maanshan	61.0000	67.2000	94.0000
Wenzhou	63.8000	70.0000	95.5000	Huangshan	69.6667	81.2500	92.7500
Huzhou	85.0000	109.7500	127.0000	average	58.5251	79.0622	102.8187

Taizhou^①^ Suzhou^③^ belongs to Jiangsu province, Taizhou^②^ belongs to Zhejiang province, Suzhou ^④^ belongs to Anhui province.

During the 11th-Five Plan, the GCA levels in Shanghai, Huaian, Suzhou^①^, Lishui, Ningbo, Hangzhou and Huzhou exceed 80 word frequency, other cities in YRD region are lower than 80 word frequency. During the 12th-Five Plan, the GCA levels in Lishui, Hangzhou, Huzhou, Huaian, Hefei, are greater than 100 word frequency, and six cities displayed an average GCA level ranging from 90 to 100 word frequency. The remaining 30 cities had an average GCA level lower than 90 word frequency.

Furthermore, during the 13th-Five Plan, the GCA levels in Nanjing, Nantong, Suqian, Changzhou, Xuzhou, Yangzhou, Yancheng, Wuxi, Huaina, Suzhou^③^, Zhengjiang, Lishui, Ningbo, Hangzhou, Shaoxing, Huzhou, Hefei Bozhou, Lu’an, Huainan, Chuzhou, Bengbu and Tongling are greater than 100 word frequency, the GCA levels in Taizhou^①^, Zhoushan, Jinhua, Suzhou^④^ and Huaibei below 90 word frequency. Overall, the GCA levels during the 13th Five-Year Plan were notably higher than that during the 12th Five-Year Plan. Consequently, there was a significant upward trend in the GCA levels of 40 cities from the YRD cities from the 11th-Five to 13th-Five Plan.

## Empirical results and discussions

### Results of GCA on urban CO_2_ emissions

This section examines the effects of population, economic factors, energy consumption intensity, GCA, and urbanization level on CO_2_ emissions in the YRD cities. The empirical results are presented in (Table 3).

**Table 3:** The results of GCA on urban CO_2_ emission in the YRD region.

Variables	(1)$I_{it}$	(2)$I_{it}$	(3)$I_{it}$
$c$	−1.6024*** (−2.9924)	−1.5054** (−2.4958)	3.1676** (2.0750)
ln $P_{it}$	0.6174*** (14.6470)	0.6416*** (14.4102)	0.6197*** (13.7786)
ln $A_{it}$	0.8766*** (25.9435)	0.9250*** (23.9459)	0.5685*** (5.2339)
ln $EI_{it}$	0.5089*** (9.6015)	0.4975*** (8.7431)	0.4018*** (8.2273)
ln $GCA_{it}$		−0.1892*** (−2.6329)	−0.2636*** (−3.5948)
ln $UD_{it}$			1.0851*** (3.9244)
Fixed effect	Year	Year	Year
$R^{2}$	0.6560	0.6620	0.6800

***,**,*Denotes the significance at the 1, 5 and 10% significance level, the number in parentheses are t statistical value.

In column (1), the results show that population size, economic development, and energy consumption intensity have significant and positive impacts on urban CO_2_ emissions at the 1% significance level. These findings indicate that larger urban populations and faster economic growth lead to increased urban CO_2_ emissions. Conversely, a reduction in energy consumption intensity contributes to a decrease in urban carbon emissions in the YRD cities.

Column (2) includes the coefficient of GCA level is -0.1892, indicating that a higher GCA level has a significant and negative influence on urban CO_2_ emissions at the 1% significance level. This suggests that increased GCA level implies greater emphasis on energy-saving and emission reduction activities, and results in a significant reduction in urban CO_2_ emissions in the YRD cities.

In column (3), the coefficient of urbanization level is 1.0851, demonstrating rapid urbanization process has a significant and positive impact on urban CO_2_ emissions at the 1% significance level. The rapid urbanization process contributes to increased urban development, significant population growth, and improved urban infrastructure. Since China’s electricity supply primarily relies on coal-fired power generation, higher levels of urbanization lead to increased electricity consumption in transportation, manufacturing, construction, productive services, and household activities, resulting in a significant rise in urban CO_2_ emissions.

### Influence mechanism of GCA on urban CO_2_ emissions

This section examines the impact of GCA on urban CO_2_ emissions via improving industrial structure upgrading and SO_2_ emission reduction policies implementation, and decreasing carbon emissions intensity in the YRD cities. The empirical results are presented in (Table 4).

**Table 4:** the results of GCA on urban CO_2_ emissions via industrial structure upgrading, carbon intensity reduction and SO_2_ emissions policies implementation.

Variables	(1)$UIS_{it}$	(2)$I_{it}$	(3)$CI_{it}$	(4)$I_{it}$	(5)$SERI_{it}$	(4)$I_{it}$
$c$	−4.3142*** (−7.7868)	2.5868* (1.8666)	6.2284*** (4.5007)	−5.8745*** (−8.8656)	−21.6774*** (−1.0e + 03)	−2.3e + 02** (−2.4082)
ln $GCA_{it}$	0.0636* (1.6686)	−0.3050*** (−4.1592)	−0.1189* (−1.9476)	−0.0771** (−1.9769)	0.0021** (2.2695)	−0.2922*** (−3.8831)
ln $UIS_{it}$		−0.3274*** (−3.4577)				
ln $CI_{it}$				1.0769*** (22.7565)		
ln $SERI_{it}$						−10.8188** (−2.4472)
ln $P_{it}$	0.1727*** (5.1005)	0.6653*** (15.7428)	−0.1542*** (−3.8338)	0.8675*** (27.4345)	0.0055*** (6.9534)	0.6717*** (14.3093)
ln $A_{it}$	0.2432*** (5.5927)	0.5948*** (5.9377)	−0.2603*** (−2.6093)	0.9722*** (21.3157)	0.0091*** (5.7580)	0.5592*** (5.1282)
ln $EI_{it}$	0.0708** (2.3348)	0.3531*** (7.3857)	0.2936*** (6.6703)		−0.0021*** (−3.0415)	0.3723*** (7.1735)
ln $UD_{it}$	−0.6851*** (−5.2880)	1.0482*** (4.0377)	0.6390** (2.5487)		−0.0169*** (−4.3392)	1.1541*** (4.1163)
Fixed effect	Year	Year	Year	Year	Year	Year
$R^{2}$	0.1950	0.6890	0.2930	0.8900	0.2530	0.6870

The annotations are consistent with those in (Table 3).

#### Industrial structure upgrading

In column (1), the coefficient of GCA is 0.0636, indicating a significant and positive effect on urban CO_2_ emissions in the YRD region at the 10% significance level. This suggests that local governments recognize the link between energy-intensive and heavily polluting manufacturing industries, and these industries result in higher levels of energy consumption and carbon emissions. Greater GCA level prioritize industrial structure upgrading and the transformation of heavy-polluting industries towards lower-carbon alternatives.

In column (2), the coefficients of GCA and industrial structure upgrading are -0.3050 and -0.3274, respectively. These coefficients demonstrate significant and negative effects on urban CO_2_ emissions in the YRD region at the 1% significance level. The rapid transition from manufacturing industries towards productive service industries leads to reduced urban energy consumption and carbon emissions. Greater GCA promotes rapid shift towards sustainable industrial structure, accelerates the fast development of green industries and the green economy. An advanced industrial structure enhances energy consumption and carbon emission efficiency, resulting in a greater reduction of carbon emissions in the YRD cities. Thereby, greater GCA further decreases urban CO2 emissions via promoting industrial structure upgrading.

#### Carbon emission intensity

In column (3), the coefficient of GCA is -0.1189, confirming that GCA has a significant and negative impact on urban carbon emissions in the YRD region at the 10% significance level. Local governments take on the primary responsibility for energy-saving, emission reduction, and optimize urban energy consumption structures. They allocate significant resources to develop energy-saving and emission reduction technologies, resulting in improved energy usage efficiency and carbon emission efficiency. Local governments promote the low-carbon transformation of energy consumption, enhance the use of renewable energy, optimize energy consumption structures, and increase the potential for energy-saving and emission reduction in the YRD cities.

In column (4), the coefficients of GCA and carbon emission intensity are -0.0771 and 1.0769, respectively. The GCA level contribute to a decrease in urban carbon emissions, while a reduction in carbon emission intensity leads to a quick decrease in urban carbon emissions in the YRD region at the 5% significance level. These results illustrate that local governments prioritize dual-carbon attention to reducing carbon emission intensity. Greater carbon emission efficiency facilitates the acceleration of CO_2_ emissions reduction in the YRD cities, which is crucial for reducing urban carbon emission in study period. Increased GCA enlarges urban CO2 emission reduction via the decline of carbon emission intensity.

#### SO_2_ emission reduction policies implementation

In column (5), the coefficient of $GCA_{it}$ is 0.0021, the GCA level produces the significant and positive impact on the implementation of SO_2_ emission reduction policies at the 5% significance level. Greater GCA strengthens `environmental laws regulation, and increase resource input to boost energy saving and emission reduction policies implementation and promote pollutants emissions control and environmental governance, and thus enhances the implementation of SO2 emission-reduction policies.

In column (6), the coefficients of GCA and $SERI_{it}$ are -0.2922 and -10.8188, respectively, and increased GCA and SO_2_ emission-reduction policies implementation produce the significant and negative effect on urban CO_2_ emissions at the 5% significance level. Increased GCA strengthens resource inputs to control sewage, SO_2_, nitrogen oxide, and pollution waste emissions, promote urban environmental governance efficiency, and thus improve the implementation of SO_2_ emission-reduction policies. Greater implementation of SO_2_ emission-reduction policies optimizes energy consumption structure, and enhances energy usage efficiency and carbon emission efficiency, and thus results in greater decline of urban CO_2_ emissions. Accordingly, larger GCA leads to greater decline of urban CO_2_ emissions via improving the implementation of SO_2_ emission-reduction policies.

### Robustness test

This section further investigates the robustness impact of GCA on urban CO_2_ emissions reduction, selects the GCA intensity, environmental governance policies implementation, peers GCA and environmental protection investment to provide related sensitivity analysis. Here environmental governance policies implementation ($EGI_{it}$) is defined as the difference between the actual and targeted environmental governance. Environmental pollutants governance includes chemical oxygen demand (COD), nitrogen oxides (NO_X_), and ammonia nitrogen emissions. Their differences between the actual emissions reduction ratio and the target emissions reduction ratio of each pollutant are calculated separately, and then calculate the implementation of pollutants emissions reduction policies using the entropy method. Their calculation formulas are similar to Formula (1). Peers GCA level is to measure the average of provincial peers GCA level exclude focal urban GCA. Environmental protection investment is defined as the amount of urban environmental protection investment. The above factors are incorporated into model 4, and their robustness results are shown in (Table 5).

**Table 5:** The robustness results between GCA and urban CO_2_ emissions reduction.

Variable	(1)$I_{it}$	(2)$I_{it}$	(3)$I_{it}$	(4)$I_{it}$
$c$	2.0275 (1.4076)	−0.2896 (−0.0939)	5.1150*** (3.9196)	4.3211*** (2.8704)
ln $P_{it}$	0.6062*** (13.2187)	0.6167*** (13.1633)	0.5725*** (12.1683)	0.5657*** (11.3822)
ln $A_{it}$	0.5992*** (5.5094)	0.4908*** (4.3699)	0.3955*** (4.1075)	0.4351*** (3.8989)
ln $EI_{it}$	0.4081*** (8.1882)	0.4065*** (7.6071)	0.2061*** (4.1734)	0.4281*** (8.3142)
ln $GCAI_{it}$	−0.3376* (−1.9003)			
ln $GCA_{it}$		−0.3104*** (−3.8168)	−0.3322*** (−4.5920)	−0.2768*** (−3.5338)
ln $UD_{it}$	0.9555*** (3.5793)	1.2999*** (4.4694)	1.5272*** (6.1266)	1.2813*** (4.5722)
ln $EGI_{it}$		−2.2511* (−1.7172)		
ln $PGCA_{it}$			−0.4221*** (−3.9924)	
ln $EPI_{it}$				−0.1481*** (−3.3190)
Fixed effect	Year	Year	Year	Year
$R^{2}$	0.6740	0.6840	0.6530	0.666

The Annotations are consistent with those in (Table 3).

In column (1), in order to reduce the impact of GCA measurement method on urban carbon emissions, the GCA intensity can decrease urban CO2 emissions in the YRD region when substitute the measurement method of GCA. This study explores the other conditions that influence the negative impact of GCA level. In column (2), the GCA level and pollutants emission-reduction policies implementation both reduce urban CO_2_ emissions at the 10%significance level. Greater implementation of environmental governance policies ` results in larger decline of urban CO_2_ emissions in the YRD region.

In column (3), the focal GCA level and peers GCA level both decrease urban CO_2_ emissions at the 1% significance level. Peers governments have self-learning abilities and imitate the dual-carbon governance behavior of neighboring governments, leading to a rapid decline in urban CO_2_ emissions in the YRD region. In column (4), the GCA and environmental protection investment also lead to the decline of urban CO_2_ emissions at the 1% significance level.

Thereby, the GCA exhibits good robustness in decreasing urban CO_2_ emissions in the YRD region, and the peers GCA, environmental governance policies implementation and environmental protection investment also reduce urban CO_2_ emissions in the YRD region, which have good robustness.

## Conclusions and policy implications

The dual-carbon attention in GWR texts is a significant mean for local governments to influence public decision-making, allocate resources, and enhance governance effectiveness. In this study, textual information analysis and the maximum reverse matching method are employed to measure the GCA level. This study examines the effects of GCA on urban CO_2_ emissions reduction in the YRD region using an expanded STIRPAT model.

The empirical results demonstrate that larger population size, higher urbanization level, rapid economic growth, and technological progress have significant positive impacts on urban CO_2_ emissions. Conversely, GCA has a significant negative effect on urban CO_2_ emissions. Specifically, greater GCA and reductions in energy consumption intensity result in greater reduction of urban CO_2_ emissions in the YRD region. Furthermore, our findings indicate that GCA facilitates the acceleration of urban industrial structure upgrading and implementation of SO_2_ emission reduction policies, and decreases the carbon emission intensity. These improvements result in a rapid reduction in urban CO_2_ emissions in the YRD region. Thereby, increased GCA further reduces urban CO_2_ emissions via enhancing industrial structure upgrading, decreasing carbon emission intensity and improving emission-reduction policies implementation. Additionally, environmental governance policies implementation, environmental protection investment and peers GCA also result in greater reduction of urban CO_2_ emissions.

Our findings have several policy implications. Firstly, greater GCA leads to a decrease in urban CO_2_ emissions. Local governments should maintain an appropriate level of dual-carbon attention intensity, focusing on energy-saving, pollutants and greenhouse gases emission reduction, as well as ecological environment governance. This will facilitate the allocation of resources towards reducing pollutants emissions, and contribute to the improvement of resource and energy allocation efficiency. Additionally, local governments encourage firms to make greater efforts in improving carbon emission efficiency, promoting energy-saving measures, and accelerating the progress of green technology.

Secondly, dual-carbon attention plays a crucial role in industrial structure upgrading, which leads to a decrease in urban carbon emissions. Local governments should prioritize the optimization of the industrial structure and energy consumption structure. They can achieve by promoting the development of clean production and green and efficient service industries. Greater efforts should be made to advance and rationalize the industrial structure, accelerate green and low-carbon transformation of the urban economy and society. Quick industrial structure upgrading will result in greater urban carbon emission reduction.

Third, dual-carbon attention contributes to a decrease in carbon emission intensity, thereby prompting a greater reduction in urban carbon emissions. Local governments should focus on reducing the proportion of coal consumption and increasing the share of renewable energy consumption in the overall energy consumption structure. Optimizing the energy consumption structure and reducing the carbon intensity of the energy sector should also be prioritized. By improving energy usage efficiency and carbon emission efficiency, local governments can significantly reduce urban carbon emissions.

Finally, local governments should improve the policy implementation of urban energy saving, emission reduction, and environmental governance. Local governments strengthen performance evaluation systems and promotion mechanisms for officials in energy conservation, emission reduction, and environmental governance constraints. Local governments can accelerate the rapid decline of urban carbon emissions by enhancing the implementation of those policies.

In summary, local governments should give careful consideration to industrial structure upgrading, energy usage efficiency, and carbon emission efficiency. By enhancing these driving factors, they can achieve significant reductions in urban carbon emissions.

## Data Availability

All data generated or analyzed during this study are included in this published article. Urban carbon emissions are source form China carbon emission accounting database (CEADs, https://www.ceads.net.cn/). The data on local governments’ dual-carbon attention were computer-generated and gathered in the experimental area; the data source related to urban development and government working reports concern to Intellectual property. They are not yet accessible to the general public or over the internet. If necessary, some data on dual-carbon attentions can be acquired from the corresponding author.
